# Supplementary figures and images for: Crude α-Mangostin Suppresses the Development of Atherosclerotic Lesions in Apoe-Deficient Mice by a Possible M2 Macrophage-Mediated Mechanism
Source: Int J Mol Sci. 2019 Apr 7;20(7):1722. doi: 10.3390/ijms20071722 (PMC6480575; doi:10.3390/ijms20071722)

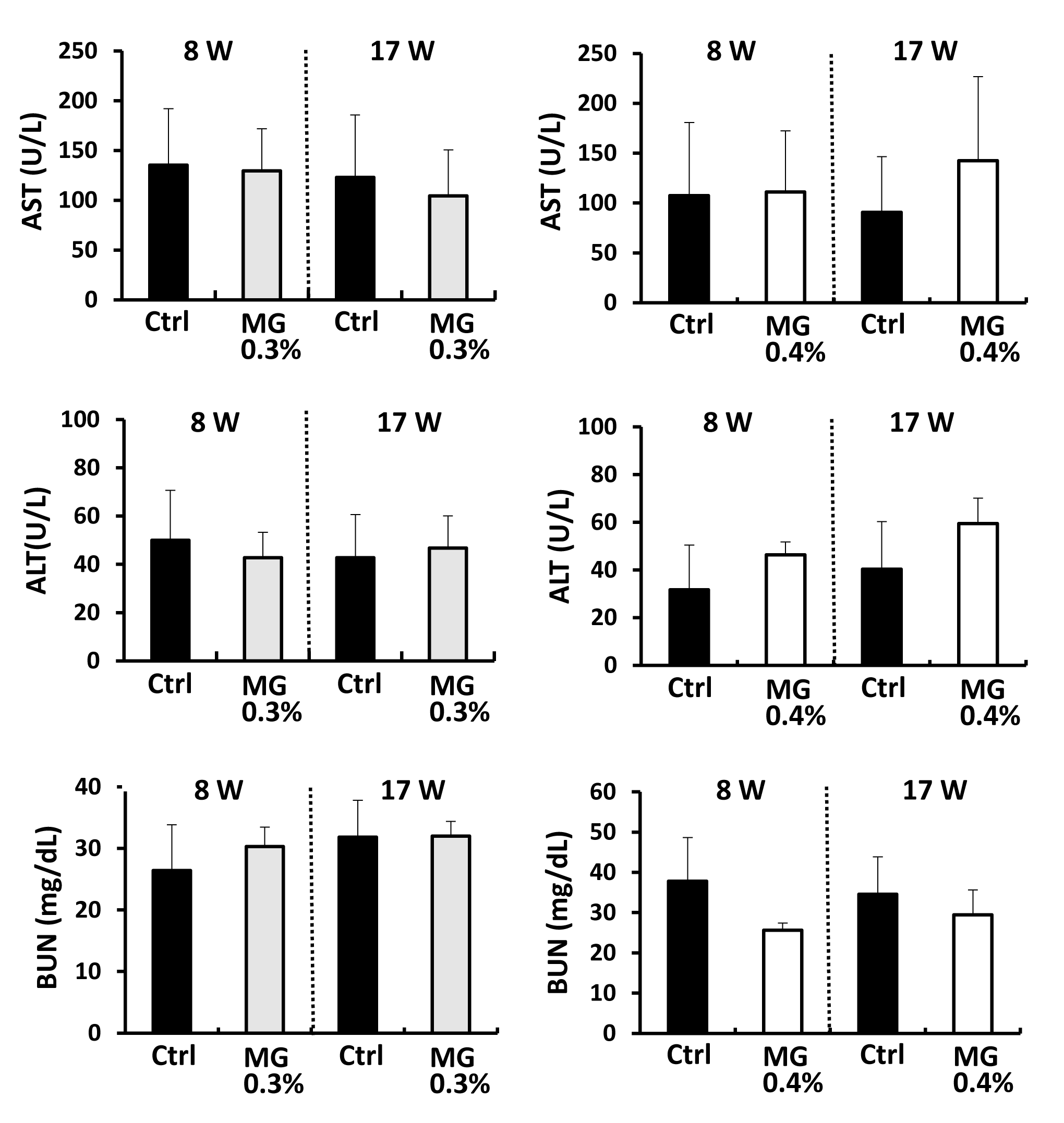

Supplement: Supplementary file 1 [file ijms-20-01722-s001.zip › ijms-458944-supplementary.tif]
